# Demonstration of Optical Coherence Tomography Guided Big Bubble Technique for Deep Anterior Lamellar Keratoplasty (DALK)

**DOI:** 10.3390/s20020428

**Published:** 2020-01-12

**Authors:** Shoujing Guo, Nicolas R. Sarfaraz, William G. Gensheimer, Axel Krieger, Jin U. Kang

**Affiliations:** 1 Department of Electrical and Computer Engineering, Johns Hopkins University, Baltimore, MD 21218, USA; jkang@jhu.edu; 2 Department of Mechanical Engineering, University of Maryland, College Park, MD 20742, USA; nrsarfaraz@gmail.com (N.R.S.); axel@umd.edu (A.K.); 3 Warfighter Eye Center, Malcolm Grow Medical Clinics and Surgery Center, Joint Base Andrews, MD 20762, USA; williamgensheimer@gmail.com

**Keywords:** common-path optical coherence tomography, distal sensor integrated needle, AUTO-DALK, deep anterior lamellar keratoplasty, big bubble technique, Descemet’s membrane

## Abstract

Deep anterior lamellar keratoplasty (DALK) is a highly challenging procedure for cornea transplant that involves removing the corneal layers above Descemet’s membrane (DM). This is achieved by a “big bubble” technique where a needle is inserted into the stroma of the cornea down to DM and the injection of either air or liquid. DALK has important advantages over penetrating keratoplasty (PK) including lower rejection rate, less endothelial cell loss, and increased graft survival. In this paper, we successfully designed and evaluated the optical coherence tomography (OCT) distal sensor integrated needle for a precise big bubble technique. We successfully used this sensor for micro-control of a robotic DALK device termed AUTO-DALK for autonomous big bubble needle insertion. The OCT distal sensor was integrated inside a 25-gauge needle, which was used for pneumo-dissection. The AUTO-DALK device is built on a manual trephine platform which includes a vacuum ring to fix the device on the eye and add a needle driver at an angle of 60 degrees from vertical. During the test on five porcine eyes with a target depth of 90%, the measured insertion depth as a percentage of cornea thickness for the AUTO-DALK device was 90.05%±2.33% without any perforation compared to 79.16%±5.68% for unassisted free-hand insertion and 86.20%±5.31% for assisted free-hand insertion. The result showed a higher precision and consistency of the needle placement with AUTO-DALK, which could lead to better visual outcomes and fewer complications.

## 1. Introduction

**Figure 1:**
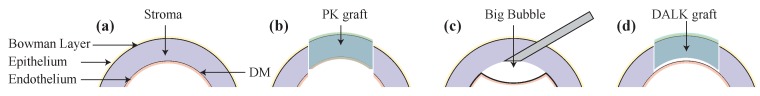
(**a**) Corneal micro-anatomical structure with epithelium, Bowan layer, stroma, Descemet’s membrane (DM), and endothelium. (**b**) Penetrating keratoplasty (PK) with full thickness corneal grafting. (**c**) Deep anterior lamellar keratoplasty (DALK) with big bubble formation. (**d**) DALK with partial thickness corneal grafting.

Blindness is a global public health issue and the worldwide blind population is estimated to be 36 million with an estimated 1.5–2.0 million new cases of unilateral blindness every year [1]. For most patients blinded from corneal diseases, the only curative treatment is corneal transplant surgery. The cornea is comprised of five layers: the epithelium, Bowman’s layer, the stroma, Descemet’s membrane (DM), and the endothelium from top to bottom as shown in Figure 1a. Penetrating keratoplasty (PK) is the main keratoplasty technique generally used. PK is a procedure in which the full thickness of the host cornea is replaced by a donor graft as shown in Figure 1b. An alternative technique is deep anterior lamellar keratoplasty (DALK), as shown in Figure 1d, where the anterior part of the cornea is replaced but the host DM and endothelium are preserved, which theoretically eliminates the potential for endothelial cell immune rejection. There are other important advantages of DALK over PK, such as lower long-term rejection rate [2], reduced rate of endothelial cell loss [3], and increased predicted graft survival [4]. DALK procedure is considered extraocular not intraocular because the procedure does not penetrate DM, which could lead to a reduced risk of intraoperative complications and postoperative complications [5].

The goal of DALK is to remove most stromal tissue, leaving only the endothelium, DM, and a small amount of residual stromal tissue. Some authors have argued that removal of all stromal tissue is the goal, which is referred to as “baring of DM” [3]. However, other authors advocate leaving a thin posterior layer of stromal tissue, sometimes referred to as Dua’s layer, in addition to the DM and the endothelium [6,7]. The most difficult part of DALK is to separate anterior stromal tissue from DM or deep posterior stromal tissue and currently, this procedure relies heavily on the visualization through a surgical microscope, microsurgical experience, and tactile feel to determine corneal depth. The most popular DALK technique uses the big bubble (BB) technique described by Anwar, where air is injected into the space between the stroma and the DM to separate the layers [8] as shown in Figure 1c. There are many modifications of the technique that report safe and effective outcomes [9,10,11]. However, inserting a needle into the cornea is a challenging step which often leads to the perforation of the cornea. The needle may produce a DM perforation, necessitating conversion to PK, if inserted too deep, or no big bubble may be formed if the air is injected too shallow. The reported average rate of perforation during DALK is 11.7% and that rate goes up to 31.8% during a surgeon’s initial learning curve [3,12]. In one study, there was a 60% rate of conversion to PK during the first 10 grafts [13].

Therefore, there is a need for novel DALK technologies that could decrease the rate of perforation and conversion to PK. The key to a successful full big-bubble formation is the precise depth control of the needle insertion. Many surgeons believe the needle must get as close as possible to DM, which could be interpreted as 90+% corneal depth without perforation [14,15,16]. Since it was first demonstrated in 1991 [17], optical coherence tomography (OCT) has emerged as a leading technology for ophthalmic visualization, especially for retinal structures [18] and been widely applied to ophthalmic surgery and research [19,20,21,22]. Compared to the time-domain approach (TD) of OCT, swept source (SS) and Fourier domain (FD) OCT techniques have superior sensitivity at the equivalent signal-to-noise ratio (SNR) as well as better axial resolution [23]. Cogliati et al. achieved 2 μm axial and lateral resolutions on the human cornea with the handheld Gabor-Domain OCT [24]. The intraoperative OCT had been applied to the DALK procedure for verification of insertion depth of the needle [25]. Kang et al. used the common-path OCT image to guide the microinjector for subretinal injection [26,27]. B-mode and C-mode OCT imaging have also been used to track various surgical tools underneath the tissue [28,29,30]. Based on these imaging techniques, a group at Duke University used a cooperative device along with real-time B-scan OCT data to assist in DALK needle insertion [31] and Shin et al. used an M-mode OCT integrated needle for lamellar keratoplasty [32]. However, none of these approaches provide an automated solution or show the ability to form a big bubble on the cornea after insertion.

In this paper, we successfully designed and evaluated the OCT distal sensor integrated needle for the big bubble technique for DALK. We also used OCT guidance for micro-control of an ocular robot, the AUTO-DALK device. This device allows for the autonomous insertion of a needle for pneumo-dissection based on the depth-sensing results from the OCT system. An earlier prototype of AUTO-DALK was introduced by the authors in [33]. In contrast, this work adds a detailed description of the OCT sensor design and integration, includes successful big bubble results, and contains a comparison to manual needle insertion under OCT guidance. The distal sensor integrated needle was tested on the ex-vivo porcine cornea to verify the sensing results during the deformation of the tissue. A comparison of expert manual insertion, expert manual insertion with OCT guidance and AUTO-DALK device insertion was conducted on five ex-vivo porcine eyes. The insertion time and final insertion depth as a percentage of cornea thickness were recorded and verified by the B-scan OCT system. To further verify the ability of the proposed fiber sensor integrated needle, it was tested on the bovine cornea to demonstrate the big bubble formation.

## 2. Methods and Materials

### 2.1. Common-Path OCT

**Figure 2:**
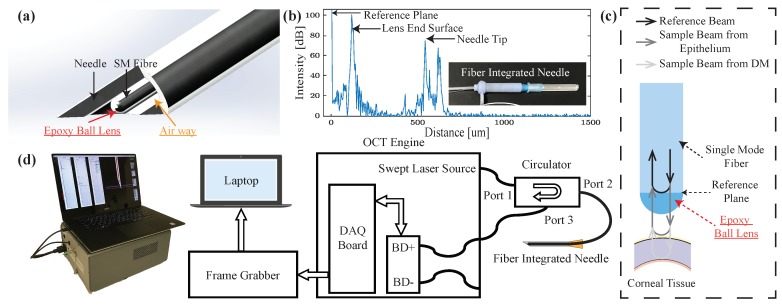
(**a**) Schematic of the distal sensor integrated needle (**b**) A-scan image of the needle tip, lens surface, and reference plane. (**c**) Schematic of the proposed ball lensed fiber probe (For simplicity, only show the sample beam from epithelium and DM). (**d**) System diagram of the common-path swept source optical coherence tomography (OCT) system (BD: balanced detector).

To acquire the depth information from the single-mode fiber (1060XP, Core Index: 1.45, Thorlabs, Newton, NJ, USA), we utilized the home-built common-path swept source OCT system (CP-SSOCT), as shown in Figure 2d. The system consists of a swept-source OEM engine (AXSUN, Billerica, MA, USA), a broadband circulator (BPICIR-1060-H6, OF-Link, ShenZhen, China), a broadband mini optical attenuator (BVOA-1050-L-10-FA, OF-Link, ShenZhen, China), a Camera-Link frame grabber (PCIe-1433, National Instrument, Austin, TX, USA) and a laptop (Precision 5520, DELL, Round Rock, TX, USA). The center wavelength of the system is 1060 nm with an output power of 2 mW, sweeping rate at 100 kHz and the maximum sensing depth in the air and the tissue is 3.7 mm and 2.8 mm, respectively. CP-SSOCT does not require a separate reference arm since the reflected beam from the end-facet of the single-mode fiber probe acts as a fixed reference beam [34]. In our case, the reflection comes from the interface of the fiber end-fact and the ball lens (see Figure 2c) with a refractive index of 1.7 and with a calculated beam power of 6.3% of the total normal incident light based on the Fresnel equations acts as the reference beam. This setup maintains the reference beam power independent of the working environment. The result interferometric signal is collected by the built-in balance detector and processed by the Camera Link DAQ board with a digitizer of sampling rate up to 500 MSPS with 12-bit resolution. Since the reference and sample beams come from the same fiber, the OCT signal cannot be distinguished from the background by the balanced detector. Thus, the single input of the balanced detector was therefore disconnected intentionally. The digitized signals were captured by the frame grabber and sent to the laptop via a high-speed Thunderbolt 3 port. To achieve the sensing speed of 100 kHz A-scan line speed, the sampled OCT spectrum data was processed in parallel with a discrete graphic card (GPU) on the laptop. The measured axial resolution was 4.5 μm inside the tissue. Additionally, both reference and sample reflected light share the same light path, which makes CP-SSOCT immune to dispersion and polarization noises. The CP-SSOCT system was packed in a benchtop electronics enclosure and make it compact and easy to integrate with other robotic or surgical systems.

### 2.2. OCT Distal Sensor Integrated Needle

As shown in Figure 2a, the distal sensor integrated needle had a minimalistic and compact structure by sharing the inner space of the needle with the fiber. The needle size in our design can be 25-gauge or 27-gauge which can provide us with the additional space for pneumo-dissection. This forward-looking OCT distal sensor can acquire the positional contexts of the forwarding tissue. By setting and calibrating the offset between the needle tip and fiber’s end surface (reference plane), the exact distance between the needle tip and the desired layer can be interpreted. To further enhance the SNR inside the cornea and protect the reference surface, a high-index epoxy ball lens is attached to the end of the single-mode fiber [35,36]. The ball lens was fabricated by applying a UV curable epoxy (NOA 170, n = 1.7, Norland, Cranbury, NJ, USA) to the fiber end, which naturally forms a semi-spherical shaped lens, owing to surface tension. The length of the lens was measured as 57.2 μm with a surface radius of 71 μm. The corresponding beam waist diameter and the Rayleigh range in the air are 8 μm and 84.5 μm, respectively [35]. The typical offset between the fiber’s end surface and the needle tip is 550 μm and this will provide us with the best protection of the fiber and less additional resistance during the insertion. The offset was controlled by a single-axis translation stage with a standard micrometer (PT1, Thorlabs, Newton, NJ, USA) and A-scan image were acquired to determine the offset as shown in Figure 1b. One thing to be noticed is that the needle tip signal in the A-scan image was from a glass slide sample which was placed right in front of the needle tip for the offset measurement and calibration. Once the offset was fixed, there is no need to image the needle tip during the experiment. As for the ball lens-end signal in the A-scan images, it is from the lens end surface and the signal was visible during the whole experiment. However, the signal of the lens-end could be misinterpreted as part of the corneal tissue during the insertion test. Thus, we removed the signal from the lens-end by subtracting it as a part of the background signal during our data processing. Once the offset distance was confirmed, the needle and distal sensor were glued together with UV-curing optical adhesives (NOA61, Norland, Cranbury, NJ, USA) at the end of the needle without blocking the airway.

### 2.3. AUTO-DALK Device Design

**Figure 3:**
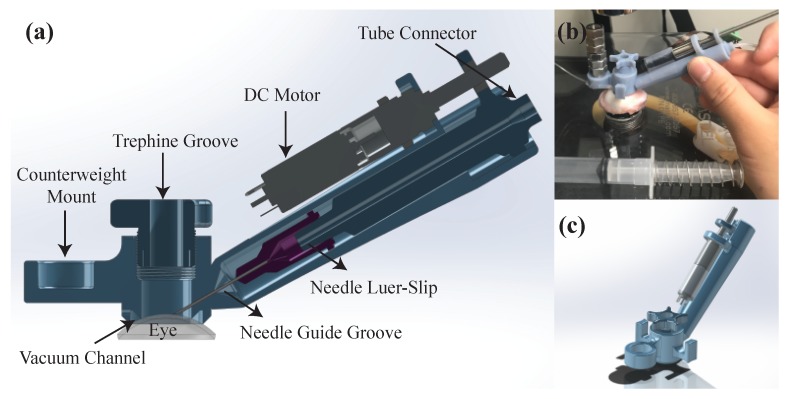
(**a**) Cross-section CAD view of the AUTO-DALK device. (**b**) Experimental setup using the AUTO-DALK device on porcine eye. (**c**) CAD isometric view of the AUTO-DALK device.

Figure 3a shows the cross-section computer-aided design (CAD) view of the proposed AUTO-DALK device. The needle insertion was fully automated by a miniature 8 mm brushed DC motor (347726, Maxon, Sachseln, Switzerland), a needle guide groove and a needle adaptor. The design is based primarily on a manual trephine, with the added ability to advance the needle to a precise depth using a motor and autonomous control loop. The motor with lead screw weighs less than 15 g. Using a pitch of 0.5 mm, gear ratio of 64:1, and 400 encoder steps (with quarter counts), the positioning resolution of the needle drive is 1.25 μm (beyond that of the OCT sensor). Based on previous research, various insertion angles have been used, such as 60°, 75° and even close to 90° from vertical [8,9,16] for visibility and improved control. Our device sets the angle to 60° to preserve a reasonable SNR of the OCT sensor since these other factors aren’t as much of a concern in the automated procedure. The device body includes a vacuum channel to fix the device onto the eye, which shares a similar design with the commercially available trephines. It also incorporates a counterweight to aid in balancing the weight of the motor. Figure 3b shows the AUTO-DALK device during testing on a porcine eye. The prototype device was designed using Solidworks and printed on a Stratasys Objet30 Pro polyjet printer using VeroBlue material. The overall weight of the AUTO-DALK device is 43.74 g including the counterweight and DC motor.

### 2.4. Signal Processing and Control Methods

**Figure 4:**
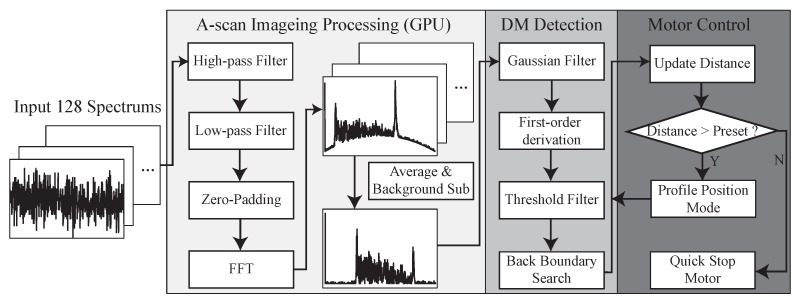
Data processing flowchart consisting of (1) OCT A-scan data processing, (2) DM detection, and (3) motor control.

Figure 4 summarizes the workflow of the proposed A-scan processing and motor control method, which contains three main modules, A-scan imaging processing, DM layer tracking, and motor control. The OCT engine returns 128-buffered spectral data every 1.28 ms and a single signal processing and control operation required 1.11 ms to obtain the position of the DM based on our measurement. These oversampled data are useful to increase the SNR due to the temporally and spatially averaged data that can effectively remove speckle noise, which is one of the main noise factors in A-scan images. Then, high-pass and low-pass filters are applied to these oversampled data to remove the DC component and high-frequency noise. Next, zero padding and fast Fourier transform (FFT) is conducted. The 128 A-scan images are averaged to one A-scan image and the background signal is subtracted. To handle these oversampled data and use it for intraoperative guidance, all these steps were implemented in GPU to significantly decrease the processing time. The final A-scan image contains 1024 data points that cover a 2771.7 μm distance inside the tissue from the end of the fiber probe.

The processed A-scan image is passed to the next step that detects the back boundary of the cornea, which is the DM and endothelium layer in our case. At this preliminary test, the AUTO-DALK device employs a fairly simple tracking and closed-loop control system. The 1 by 5 Gaussian averaging filter is used to smooth the A-scan image and reduce the noise. To apply the filter across the whole A-scan image, this was achieved by convolving the 1D Gaussian distribution with the original A-scan image and Equation (1) described the filtered signal intensity ad each pixel location. Here, x[n] stands for corresponding A-scan image intensity at pixel location of *n*, *y* is the A-scan image after filtering and *h* is the Gaussian filter with the length of 2M+1.

(1) $$y\left[n\right]=\sum_{m=n-M}^{n+M}h\left[n-m+M\right]x\left[n\right].$$

Then, we apply first-order derivation to the filtered A-scan image, which can be described as Equation (2). We threshold the result and search backward to determine the boundary. The calculated distance in the A-scan image represents the distance between the back boundary to the reference plane. To acquire the distance between the needle tip and back boundary, we subtract the needle offset from the tracking result, which has been pre-loaded into the program and this provides the real-time distance from the DM.

(2) $$\frac{dy}{dn}=y\left[n+1\right]-y\left[n\right].$$

This position information is updated and fed into the control loop. The updated measurement is compared with the preset distance, which determines how close the needle should get to the DM, usually around 100 μm on the porcine cornea. If the distance calculated from the tracking algorithm is larger than the preset value, the calculated position value will be transmitted to the next step for the motion control. Here, we used the motor controller’s build-in profile position function to control the motor. This mode works in such a way, when we input the position, it will start moving at the pre-set speed. If any new position was sent during this process, the motor will use the new position instead. Combining with the quick stop function, we can control the motor at the desired speed and make it stop once the preset distance to the DM is reached. A customized real-time user interface is designed and programmed through C++ and C# to let the user control and monitor the whole insertion procedure.

## 3. Experiments and Results

### 3.1. Deformation Test

**Figure 5:**
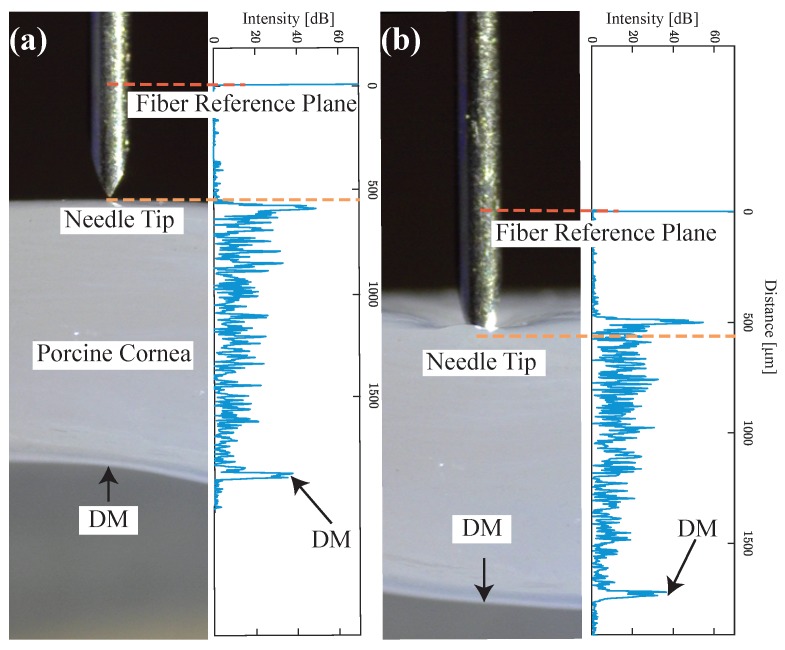
A-scan images (right side in (**a**) and (**b**)) and corresponding microscope cross-sectional image (left side in (**a**) and (**b**)) of cornea. The distance between the needle tip and the OCT sensor was pre-calibrated as 550 μm, (**a**) when the needle is just above the cornea, (**b**) when the needle tip starts to pierce the cornea and causing maximum deformation.

To test the proposed sensor integrated needle’s ability to track the tissue layer during deformation, the experiment was conducted on ex-vivo porcine eyes, purchased from Wagner’s Meat in Mt Airy, MD. The test was done on the same day that the eyes were harvested. For better visualization, the cornea was carefully separated from the eye and fixed on a special cornea holder. The holder was designed based on the commercially available cornea holder with an additional opening on the side to be able to image the cross-sectional view of the cornea. This will allow us to match the A-scan image with the physical image of the cornea. The holder was 3D printed on an uPrint SE Plus ABS printer and it can hold the cornea in position during the test and preserve the normal curvature of the cornea. The needle was fixed on a vertical translational stage to approach the cornea precisely and the offset between the needle tip and OCT sensor reference plane was set to 550 μm. A-scan images (right side of Figure 5a,b) and the corresponding microscope cross-sectional image (left side of Figure 5a,b) of the cornea are shown to easily identify the cornea layers corresponding to the peaks in OCT A-scan images. Figure 5a corresponds to when the needle is just above the cornea, clearly showing the A-scan of whole cornea layers including DM. Figure 5b corresponds to when the needle tip starts to pierce the cornea, causing the maximum deformation of the cornea. The forward-looking OCT sensor clearly shows all the peaks corresponding to the corneal layers even when the tissue is highly deformed and the measured distance between the needle tip and DM can represent the real value.

### 3.2. Unassisted and Assisted Freehand Insertion Test

**Figure 6:**
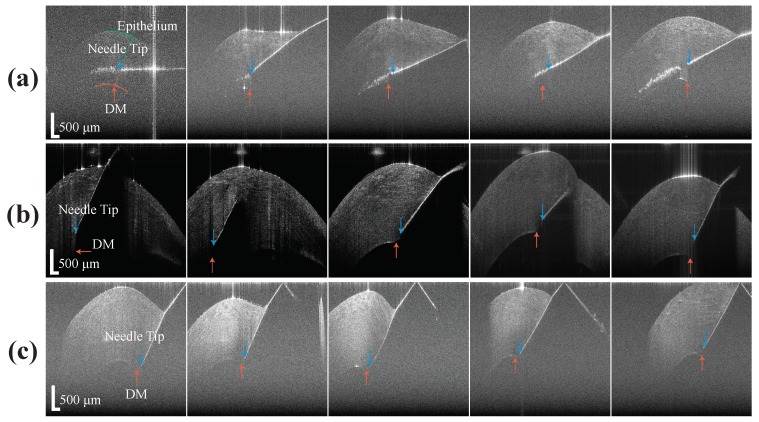
The B-scan of the porcine cornea with needle tip inside, (**a**) results from freehand test, (**b**) results from assisted freehand test, (**c**) results from the AUTO-DALK test.

To compare unassisted and assisted freehand insertion, the experienced surgeon performed the test with a normal 27 G needle and a 25 G distal sensor integrated needle on five porcine eyes for each test. The reason for the use of porcine cornea is because it shares many biomechanical properties with human cornea [37]. The freehand test was made to closely mimic the conditions of the clinical standard DALK procedure. For better visualization purposes, the surgeon injected a small amount of air into the anterior chamber (AC) before the insertion and injected a small amount of air into cornea after it. Also, the surgeon performed the unassisted freehand test through a 10× microscope. All these additional steps were not done in other test groups. In the experimental setup, eyes were held in place using suction on a Mastel Practice Eye Mount. Suction was applied to each eye by drawing air from the attached syringe, before clamping down the vacuum tube. The Intraocular pressure (IOP) of the eyes was verified tactilely by an ophthalmologist, based on firmness, to roughly approximate that of a normal eye. However, the IOP remains slightly above normal due to suction mounting. As for the assisted freehand insertion, the needle was inserted with the assistance of the CP-SSOCT system and the A-scan images were taken continuously to determine the distance between DM and the needle tip. To better evaluate and compare the result, we used a high-speed in-house built OCT system to acquire B-scan images of the cornea with the needle still stayed at the final position. As shown in the first image of Figure 6a, the epithelium (green curve) and DM (orange curve) were manually segmented for depth estimation purposes. The stroma above the needle and the stroma below the needle were segmented based on the needle position and the final insertion depth as a percentage of cornea thickness can be calculated. Additionally, the time of insertion was recorded for further comparison. The example video clip for the A-scan images acquired during the assisted freehand test was included in the supplementary material.

### 3.3. AUTO-DALK Insertion Test

**Figure 7:**
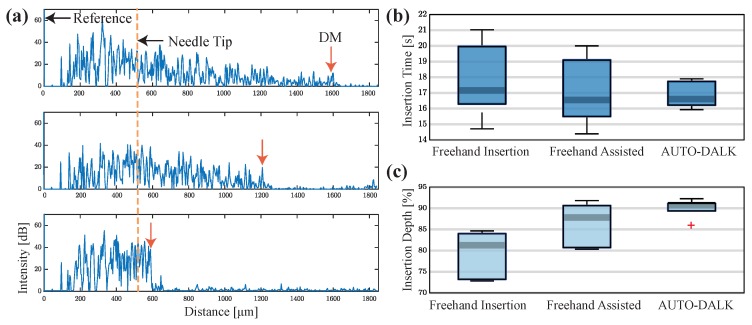
(**a**) A-scan images (inserted at 60 degrees from vertical) of the porcine cornea as the needle advances toward DM. Distance between the needle tip and DM, 1100 μm (**top**), 700 μm (middle), and 120 μm (**bottom**). (**b**) Comparison of the insertion time. (**c**) Comparison of the insertion depth as a percentage of cornea thickness.

To validate the performance of the AUTO-DALK device, a similar insertion test was performed. One thing to notice is that the porcine eyes used for testing the AUTO-DALK device was two days after the eyes were harvested due to the problem of logistics. This resulted in the thickness of the eyes in these groups increased 100 to 200 μm and total thickness ranged from 1.1 mm to 1.5 mm. Thus, we preset our target distance to the DM at 120 μm and this will give us above 90% insertion depth for all the eyes. Due to the weight of the device, the suction mechanism didn’t provide enough holding force to fixate the device on the eye, requiring manual stabilization during the insertion test. During the procedure, the relative position between the eye and the AUTO-DALK device was stable and no obvious movement was noticed. The preset offset distance was 550 μm in all the 25 G sensor integrated needle we made and the motor speed was set to the lowerest value at this test to better monitor the insertion and reduce the risk of perforation. The needle tip offset and target distance was input into the control software before the device was mounted on the cornea. The timer started when the device was on the cornea and stopped at the moment that A-scan images stabilized after the motor stopped moving. The B-scan image was taken through the trephine groove while the device is still on the eye as Figure 6c and the depth was analyzed by the same method mentioned above. Figure 7a shows the A-scan images during one representative AUTO-DALK insertion test, which inserted at 60 degrees from vertical. The top image shows the needle tip penetrating the cornea where the distance between the needle tip and DM was approximately 1100 μm. As the needle advanced, the DM peak moves closer to the needle tip to the distances of around 700 μm (middle) and 120 μm (bottom), respectively. The video of the A-scan images during the insertion was included in the supplementary material.

### 3.4. Comparison of Needle Insertion Tests

The results from the three test groups were plotted in Figure 7b,c to compare the insertion time and depth accuracy. Comparing the time to finish the insertion, the surgeon used 17.75 s ± 2.48 s in unassisted test and 17.11 s ± 2.25 s in the assisted test. In the AUTO-DALK test, the device used 16.88 s ± 0.86 s. Although the difference between the average time to finish the insertion task was small between the three test groups, the standard deviation indicates the performance of the unassisted and assisted freehand is similar, but the AUTO-DALK device was more consistent. Comparing the insertion depth between unassisted and assisted freehand tests, the surgeon improved the performance from 79.16% ± 5.68% to 86.20% ± 5.31% with the assist of the distal sensor integrated needle. The AUTO-DALK device accomplished the needle insertions with an average depth of 90.05% ± 2.33%, significantly higher than the other two manual insertion results. The standard deviations also indicate the AUTO-DALK device was more consistent in the insertion depth. Due to the relatively small sample size, we performed the nonparametric Wilcoxon rank-sum test to further evaluate our results, which is equivalent to a Mann-Whitney U-test. The calculated *p*-value was p=0.0079 and *h*-value was h=1. This indicated the rejection of the null hypothesis of equal medians at the default 5% significance level, which suggested the insertion depth with AUTO-DALK was statistically significantly deeper compared to freehand.

### 3.5. Feasibility Test of Pneumo-Dissection on Bovine Eyes

Once the needle was inserted into 90% above the thickness of the cornea during the standard DALK procedure, the air will be injected to separate the DM from the stroma to form the big bubble. However, during our tests on porcine eyes, the big bubble could not be achieved even when the insertion depth was above 90% of the cornea depth. The possible reason for this was that the porcine cornea we acquired from the local slaughterhouse exhibited cornea clouding indicating significant endothelium cell loss [38,39]. Also, the typical slaughter age for pigs is 5–6 months and at that age, the DM and endothelium aren’t fully developed yet. These will lead to the failure of big bubble formation on such eyes. To verify the pneumo-dissection ability of the proposed distal sensor integrated needle, a separate proof-of-concept experiment was conducted on bovine corneas with an assisted insertion method. The bovine cornea is not suitable for the insertion test due to its significant size and since the bovine corneal tissue we used was much tougher to penetrate compared to the porcine and human cornea. However, the bovine eyes we purchased (Old Line Meats, Baltimore, MD, USA) have the typical slaughter age of 1.5 years and the DM has been fully developed, which makes it suitable for this big bubble test.

**Figure 8:**
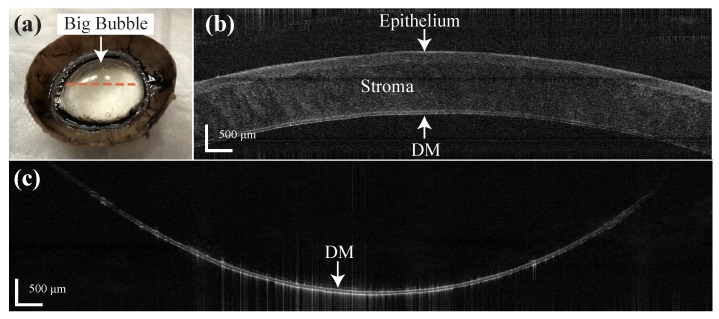
(**a**) Big bubble formation result by the distal sensor integrated needle on the bovine cornea. (**b**) B-scan of the bovine cornea before the insertion (epithelial side up). (**c**) B-scan of the big bubble (endothelial side up) on the labeled location (orange dotted line) in (**a**).

This feasibility experiment was conducted on five bovine eyes consecutively with the assisted manual insertion. The insertion depth was recorded as 93.01%, 90.12%, 92.41%, 90.81% and 82.84%, respectively. A 10 ml syringe was connected to the needle for air injection due to the larger size of the bovine cornea. Big bubbles were achieved and DM was fully separated as Figure 8a in the three eyes with 92.41%, 93.01% and 90.12% insertion depth. The B-scan image was taken before the needle insertion with epithelium side up as Figure 8b and the total thickness of the DM and endothelium was measured to be 68.81 μm. The B-scan image of the big bubble was taken with the endothelial side up (Figure 8c) and the thickness of the DM and endothelium was 60.08 μm. This result indicates the stroma was fully separated from the DM by the distal sensor integrate needle. In the case of insertion depth as 90.81%, the DM was only separated around the center part of the cornea and formed a partial big bubble. The air leakage from the DM and endothelium during the injection was noticed in this case and probably due to the endothelium cell loss of this cornea. The DM was not fully separated with a small amount of stroma left on it in the case of 82.84% insertion depth and this was because of the shallow insertion depth of the needle.

## 4. Discussion and Conclusions

In this paper, we successfully implemented the OCT distal sensor integrated needle and demonstrated the AUTO-DALK device for autonomous needle insertion on the porcine cornea. As a significant determiner of pneumo-dissection success [16], the AUTO-DALK device performed needle insertions with an average depth of 90.05% ± 2.33% without perforation and this improvement have large clinical ramifications. Also, the freehand insertion outcome can be improved by the distal-sensor integrated needle according to the comparison of our tests. The shared path design of the needle and the distal sensor can track the DM during tissue deformation and can inject the air to form the big bubble. This feasibility study leads us to believe the distal sensor integrated needle with a micron-accuracy robot can improve overall DALK successful rate.

Compared to the previous M-mode OCT integrated needle approach [32], they proved the sensor integrated needle has the ability to continuously track the DM during the free-hand insertion. However, the needle still has to be placed by hand, which the final position accuracy will suffer from the physiologic tremor and this will eventually lead to poor positioning accuracy and a waste of the accuracy of the OCT system. The cooperative device along with real-time B-scan OCT introduced by Duke University [31], has addressed the positioning accuracy problem by using a robot arm to assist the insertion procedure. However, it required for strict alignment between the intraoperative OCT and the robot to be able to keep tracking the needle tip inside the corneal tissue. Also, the depth sensing rate will be much lower compared to our approach, due to the massive computing required for image/volume processing. Our approach provides a more compact and faster-tracking solution. Integrated with 3D-printed AUTO-DALK tools, the overall size and weight are significantly smaller compared to the robotic arm, which could be easily deployed in the operating room. Although five porcine eyes for each test group is a relatively small sample size compared to other studies, the difference between the test groups was striking and statistically significant.

Notably, there are some limitations to this work. Due to the weight of the device and air leakage at the eye interface of the device, the suction mechanism did not provide enough holding force to fixate the device on the eye, requiring manual stabilization during insertions. One might expect a decreased perforation rate in the assisted freehand insertion and AUTO-DALK trail, however, we only used five eyes for each test and there was no perforation in our case. Also, the average time used to complete the needle insertions for the three groups was similar. One reason for the relatively slow speed for the autonomous insertions was that the motor was set to the lowest speed for better monitoring during this feasibility study. Another reason is during the initial insertion, the tracking algorithm did not work as well due to relatively low SNR of the DM layer on the A-scan image and lower speed can help us avoid perforations. Lastly, the ultimate proof of success is autonomous needle insertion and full pneumo-dissection, which we had to test separately due to limitations of the tested eyes.

The future work will be focused on designing a more compact device, improving the suction, and reducing the weight for the whole device. Currently, we used a DC brushed motor for the insertion task and the size and weight of this motor took a great part in the total size and weight. This could be lower by using a compact motor or the advanced piezo motor. With the decreased total weight, the suction will be improved and the main body of the device can be printed in a glossy style to improve the airtightness at the interface. Also, signal processing and control methods can be further improved by including PID control and data filtering methods to achieve faster insertion speed and reduce depth variability. Although PID control requires significant computing power for the integrative part and this could slow down the whole system speed, this problem can be addressed by using GPU for data processing. Another possible method could be the use of a Kalman filter, which has been applied to the previous CP-SSOCT system for motion compensation and has the ability to catch up with the sensing speed [26]. Future experiments will include tests on the human cadaveric cornea with a larger sample size and evaluation of the final big bubble formation results.

In summary, our AUTO-DALK platform offers real-time distal sensing and improved needle insertion depth in a porcine eye DALK model. We believe this can be used for improving the DALK success rate and thereby lower the graft rejection rate. This could lead us to a more efficient and economical cornea treatment in the future.

## Supplementary Materials

The following are available online at https://www.mdpi.com/1424-8220/20/2/428/s1, Video S1: Ascan during AUTO-DALK and assisted free-hand test.

## Abbreviations

The following abbreviations are used in this manuscript:

DM	Descemet’s membrane
PK	Penetrating keratoplasty
DALK	Deep anterior lamellar keratoplasty
BB	Big bubble
OCT	Optical coherence tomography
TD	Time domain
SS	Swept source
FD	Fourier domain
SNR	Signal to noise ratio
CP-SSOCT	Common-path swept source optical coherence tomography
BD	Balanced detector
CAD	Computer-aided design
GPU	Graphic processing unit
FFT	Fast Fourier transfer
AC	Anterior chamber
IOP	Intraocular pressure
